# Supplementary material for: ProteinShader: illustrative rendering of macromolecules
Source: BMC Struct Biol. 2009 Mar 30;9:19. doi: 10.1186/1472-6807-9-19 (PMC2672931; doi:10.1186/1472-6807-9-19)
Supplement: Additional file 1 — ProteinShader program without source code. This compressed file contains the complete ProteinShader program including associated libraries, but no source code. A README.txt file gives an overview of the ProteinShader distribution, and the index.html file in the help subdirectory has directions on getting started with the program as well as a set of tutorials. [file 1472-6807-9-19-S1.zip › ProteinShader-beta-0_9_4-binary/help/api/org/proteinshader/gui/components/controlpanel/package-tree.html]

org.proteinshader.gui.components.controlpanel Class Hierarchy (ProteinShader API)


|  |  |  |
| --- | --- | --- |
| **Overview** \| **Package** \| Class \| Use \| **Tree** \| **Deprecated** \| **Index** \| **Help** | |  |
| **PREV**   **NEXT** | **FRAMES**    **NO FRAMES**     **All Classes** |  |


---


## Hierarchy For Package org.proteinshader.gui.components.controlpanel

**Package Hierarchies:**: All Packages

---

## Class Hierarchy

- java.lang.**Object**
  - java.awt.**Component** (implements java.awt.image.ImageObserver, java.awt.MenuContainer, java.io.Serializable)
    - java.awt.**Container**
      - javax.swing.**JComponent** (implements java.io.Serializable)
        - javax.swing.**JPanel** (implements javax.accessibility.Accessible)
          - org.proteinshader.gui.components.controlpanel.**AntialiasingPanel**- org.proteinshader.gui.components.controlpanel.**AtomScalePanel**- org.proteinshader.gui.components.controlpanel.**CartoonSideChainsPanel**- org.proteinshader.gui.components.controlpanel.**ColorPanel**
                  - org.proteinshader.gui.components.controlpanel.**AtomColorPanel**- org.proteinshader.gui.components.controlpanel.**CartoonColorPanel**
                  - org.proteinshader.gui.components.controlpanel.**ControlPanel**- org.proteinshader.gui.components.controlpanel.**DecorationsPanel**- org.proteinshader.gui.components.controlpanel.**ModifierPanel**- org.proteinshader.gui.components.controlpanel.**MotionPanel**- org.proteinshader.gui.components.controlpanel.**OptimizePanel**- org.proteinshader.gui.components.controlpanel.**RadioPanel**- org.proteinshader.gui.components.controlpanel.**SelectorPanel**- org.proteinshader.gui.components.controlpanel.**VisibilityPanel**
                                  - org.proteinshader.gui.components.controlpanel.**AtomVisibilityPanel**- org.proteinshader.gui.components.controlpanel.**CartoonVisibilityPanel**

## Enum Hierarchy

- java.lang.**Object**
  - java.lang.**Enum**<E> (implements java.lang.Comparable<T>, java.io.Serializable)
    - org.proteinshader.gui.components.controlpanel.**RadioPanel.Type**

---


|  |  |  |
| --- | --- | --- |
| **Overview** \| **Package** \| Class \| Use \| **Tree** \| **Deprecated** \| **Index** \| **Help** | |  |
| **PREV**   **NEXT** | **FRAMES**    **NO FRAMES**     **All Classes** |  |


---

# *Copyright © 2007-2008*
